# Exploring the role of gender and gendered pain expectation in physiotherapy students

**DOI:** 10.1080/24740527.2019.1625705

**Published:** 2019-06-21

**Authors:** Marudan Sivagurunathan, Joy MacDermid, Joseph Chien Yee Chuang, Allyssa Kaplan, Stephanie Lupton, Deidra McDermid

**Affiliations:** a Department of Health and Rehabilitation Sciences, Health and Rehabilitation Sciences, Western University, London, Ontario, Canada; b School of Physical Therapy, Western University, London, Ontario, Canada; c School of Rehabilitation Sciences, McMaster University, Hamilton, Ontario, Canada

**Keywords:** physical therapists, gender, gender roles, pain, quantitative

## Abstract

**Introduction**: Gender and gender role pain expectations may influence how health care providers interact with and manage their patients’ symptoms.

**Purpose**: The purpose of this study was to describe gendered traits and gender role pain expectations among physical therapy students.

**Method**: A survey assessing gendered traits and gender role expectations in relation to pain was completed by a sample of 171 physical therapy students (120 women, 51 men). Data were analyzed using descriptive statistics and differences between men and women were tested with chi-square or Kruskal-Wallis.

**Results**: Men and women in physical therapy training were not different on 13 out of 16 of the gendered traits. The exceptions were that men rated themselves as more “decisive” compared to women (mean rank = 103.8 vs. mean rank = 78.4, *P* = 0.001) and women rated themselves as more “emotional” (mean rank = 91.95 vs. mean rank = 72.01, *P* = 0.009) and more “nurturing” (mean rank = 90.89 vs. mean rank = 72.91, *P* = 0.020).

No significant differences were found in terms of gendered expectations of pain sensitivity, endurance, or in terms of personal experience of pain between the men and women in the sample. However, the majority (75%) of participants reported that women were more willing to report pain compared to men. Finally, both groups rated themselves as no different in handling pain compared to a typical man or woman.

**Conclusion**: In conclusion, men and women in training to be physical therapists demonstrate similar gendered trait profiles and little gender bias in relation to pain expectations.

## RÉSUMÉ

**Introduction**: Le sexe des prestataires de soins et leurs attentes à l’égard du rôle des hommes et des femmes en ce qui concerne la douleur peuvent influencer la façon dont ils interagissent avec les patients et prennent en charge leurs symptômes.

**But**: Le but de cette étude était de décrire les caractéristiques sexospécifiques et les attentes à l’égard du rôle des hommes et des femmes en ce qui concerne la douleur chez des étudiants en physiothérapie.

**Méthode**: Un sondage évaluant les caractéristiques sexospécifiques et les attentes à l’égard du rôle des hommes et des femmes en ce qui concerne la douleur a été mené auprès d’un échantillon de 171 étudiants de physiothérapie (120 femmes, 51 hommes), Les données ont été analysées à l’aide de statistiques descriptives tandis que les différences entre les hommes et les femmes ont été vérifiées à l’aide du test du chi carré ou du test de Kruskal-Wallis.

**Résultats**: Les hommes et les femmes étudiant la physiothérapie n’étaient pas différents pour 13/16 des caractéristiques sexospécifiques. Les exceptions étaient que les hommes se disaient plus « déterminés » comparativement aux femmes (valeur moyenne = 103,8 comparativement à valeur moyenne = 78,4, *P* = 0,001), tandis que les femmes se considéraient plus « émotives » (valeur moyenne = 91,95 comparativement à valeur moyenne = 2,01, *P* = 0,009) et plus « attentionnées » (valeur moyenne = 90,89 comparativement à valeur moyenne = 72,91, *P* = 0,020).

Aucune différence significative entre les hommes et les femmes faisant partie de l’échantillon n’a été observée en ce qui concerne les attentes sexospécifiques à l’égard de la sensibilité ou de l’endurance à la douleur, ou l’expérience personnelle de la douleur. Toutefois, la majeure partie des participants (75%) ont déclaré que les femmes étaient plus disposées que les hommes à signaler la douleur. Finalement, les deux groupes ont déterminé qu’ils ne géraient pas la douleur différemment de l”homme-type ou de la femme-type.

**Conclusion**: En conclusion, les hommes et les femmes qui étudient pour devenir physiothérapeutes présentent des profils aux caractéristiques sexospécifiques similaires et ont peu de préjugés sexistes relativement aux attentes concernant la douleur.

## Introduction

Sex and gender are two variables that play an important role in our lives.^1^ However, despite the importance of sex and gender, research on their impact on various facets of our lives are limited. With respect to health care, sex and gender have received little attention in research or training. For example, a systematic review of 1303 studies by Mansukhani et al.^2^ found that sex of the subjects was not reported by 17% of manuscripts published by the top five American nonspecialty surgical journals. The importance of considering sex and gender in health research has recently become more predominant, as indicated by the Sex and Gender Equity in Research Guidelines that encourage considering sex and gender throughout all stages of research.^3^ However, issues regarding the definition of the concepts of *sex* and *gender* and our lack of understanding about the relationship between the two concepts continue result in some confusion.^1,4^ To date most studies on gender and sex treat them as largely binary concepts. However, researchers have started to acknowledge the nonbinary nature of both sex and gender.^4,5^ Additionally, Hart et al.^4^ noted that studies do not measure gender (man/woman) and sex (male/female) separately and treat both concepts as synonymous. Though sex and gender may be interrelated, their impact on health outcomes may differ.^1^ The Institute for Gender and Health defines gender as “socially constructed roles, relationships, behaviours, relative power, and other traits that societies ascribe to women and men, p. X,”^6^ whereas sex can be defined as “biological and physiological characteristics that distinguish females from males, p. X.”^6^

Though there have been numerous studies on sex and pain,^7^ studies on the how gender role influences pain perception are limited.^8^ Gender expectations can influence how individual experience pain or behave in response to it or how people interpret the pain reports or behaviors of others. Defrin et al.^8^ suggested that gender role expectations related to pain, as influenced by learned masculine and feminine gender roles, might be better predictors of differences in pain perception than sex. Studies examining gender expectations of pain show that there are significant differences in how each sex views their own gender and the opposite gender, which reinforces the hypothesis of gender bias. For example, studies^8,9^ have found that both sexes perceived the typical man to be less sensitive and less willing to report pain than the typical woman. When looking at pain endurance, males perceived that they were similar to typical women, whereas females perceived that they were less able to endure pain than typical men.^8^

Stereotypical gender role expectations of pain may predict pain reporting, pain threshold, and pain endurance behaviors in individuals.^10,11^ For example Wise et al.^10^ found scores on the Gender Role Expectations of Pain (GREP) questionnaire to be significant predictors of pain thresholds, explaining 7% of the variance, with those who reported greater willingness to report pain compared to a “typical man” having lower pain threshold times in comparison to those who reported less willingness to report pain compared to a typical man. Additionally, GREP scores accounted for 11% of the variance in pain tolerance, with subjects who reported being less willing to report pain also having higher pain tolerance to thermal stimuli.^10^ Finally, GREP scores accounted for 21% of the reported pain unpleasantness.^10^ A similar study by Alabas et al.^11^ found that GREP score was a mediator of the sex differences in pain threshold and pain endurance.

Gender and gender role expectations of health care providers can be shaped by the nature of the health problem as well as social context and position, training, and the evidence on sex/gender in the literature. This in turn may positively or negatively influence how they interact with and manage their patients. Studies have examined how gender-related expectations of pain may influence treatment decisions among physicians,^12,13^ nurses,^12,14^ and dentists.^13,15^ These gender biases and expectations impact treatment decisions by health care providers.^13,16,17^ For example, a study by Schäfer et al.^13^ found that health care providers judging chronic back pain in patients judged men as having more pain than women and women to be exaggerating their pain compared to men. Additionally, health care providers prescribed more opioids and nonopioid analgesics to men compared to women.^13^

Given that one of the core tenets of the health profession is equality, it may be argued that sex or gender disparities might arise due to unconscious social biases. Verdonk et al.^18^ noted that gender awareness among heath care practitioners contributes to “equity and equality in health, p. 135.” Gender sensitizing programs have been shown to have positive attitude change and an empowering effect on students.^18^ As such, it is imperative to examine biases that may be present among physical therapy (PT) students. If physical therapy students hold inappropriate gender biases that persist beyond their clinical education, it may negatively impact their ability to manage pain in their patients. Studies on the relationship between education and attitude regarding persons with disabilities among PT students have found education to have a positive impact.^19^

Most of the studies to date examining gender bias in health professions have focused on physicians. PTs commonly manage patients with pain but have some important professional differences from physicians. PTs have been traditionally placed in a less “powerful” position in the health system compared to physicians. This may influence the gender profiles attracted to the profession or their training experiences. Additionally, whereas medicine has traditionally been more male dominated, PT has traditionally been a female-dominated profession, although the representation of men is more substantial in PT than in other health professions such as nursing or occupational therapy. Gender distributions within professions likely affect professional norms. Further, PTs may spend more time with each individual patient during their clinical interactions, which affects the nature and impact of the gender biases that develop during training. Given these considerations, the purpose of this article was to describe the gender role expectations of pain among PT students.

## Methods

### Recruitment

The University of Western Ontario provided ethics approval for this study. Participants were recruited from two universities located in southern Ontario. One of the co-authors (J.M.) distributed the paper version of the Gender, Pain and Expectations Scale (GPES) to first and second-year students in their master of physical therapy program, with participation in the survey being voluntary in nature. Further, a mass email invitation with a link to the online version of the survey was sent by the school and PT group e-lists.

### Participants

The final study consisted of 171 first- and second-year master of physical therapy students from two universities located in southern Ontario; the majority (70%) were women. For complete demographic data, see Table 1.

**Table 1.** Participant demographics.

Characteristics	*n* (%)
Age	
20–22	11 (6.4)
23–25	113 (65.7)
26–28	29 (16.9)
29–31	5 (2.9)
>31	5 (2.9)
Missing	9 (5.2)
Gender	
Men	51 (29.8)
Women	120 (70.2)

### Measures/questionnaire

The Gender, Pain and Expectations Scale (GPES) used in this study was developed to evaluate gendered traits and related pain expectations. Two versions of the survey were distributed. The initial survey consisted of four primary questions on gender, pain, and pain expectations. The Gender Personality Traits section of the survey consisted of 16 traditionally gendered traits, seven of which (“independent,” “aggressive,” “gentle,” “leader,” “competitive,” “sensitive,” “decisive”) were adapted from the Bem Sex Role Inventory (BSRI), considered by the researchers to be either masculine or feminine. The other nine traits (“emotional,” “confident,” “weak,” “tough,” “giving,” “accepting,” “determined,” “nurturing,” “patient”) were adapted from the literature on gendered traits that were seen as relevant to clinical practice and gender role expectations. Participants were asked to rate how likely they felt the adjectives described themselves using a 5-point scale ranging from *not at all* to *extremely*. Gendered Pain Expectations section consisted of participants rating which gender is more sensitive to pain, can endure pain, and more likely to report pain. Each factor was rated on a nominal scale with the following options: *men a lot more; men a little more; there is no difference; women a little more*; or *women a lot more*. The Pain Trait section of the survey consisted of three questions on which respondents rated themselves on pain sensitivity, endurance, and willingness to report pain on a 5-point scale ranging from *not at all* to *extremely*. Finally, on the Gender Expectations Alignment section of the survey, participants were asked to rate how well they handle pain compared to a “typical man” and a “typical woman” rated on a 3-point nominal scale with the options *better, no difference*, and *worse*. An updated version of the survey removed the adjectives *aggressive* and *weak* and the Gender Expectations Alignment section of the survey only asked participants, “How do well do you think you manage pain compared to other people of your gender?”

### Data analysis

All statistical analyses were performed using SPSS version 24.0 (SPSS Inc., Chicago, IL). Data were checked for normality of distribution and outliers. Descriptive analysis was performed on all four aspects of the survey. Due to fact that the 16 gendered personality traits and pain traits were rank-ordered Likert items, Kruskal-Wallis analysis was used to examine whether there were gender differences in how participants rated themselves on the 16 adjectives and their self-reports of pain experience. Chi-square analysis was used to examine the gender differences in gendered pain expectations and gender expectations alignment.

## Results

### Gendered personality traits

Kruskal-Wallis tests were conducted to examine the effect of gender on the 16 adjectives. Of the 16 gendered personality traits, only three were found to be significantly different between men and women: emotional, nurturing, and decisive were found to have significant gender differences (*P* < 0.05). Women rated themselves as more emotional (mean rank = 91.95) in comparison to men (mean rank = 72.01, *H* = 6.780, *P* = 0.009). Additionally, compared to men (mean rank = 72.91), women rated themselves as more nurturing (mean rank = 90.89, *H* = 5.432, *P* = 0.020). Conversely, men rated themselves as more decisive (mean rank = 103.81) in comparison to women (mean rank = 78.43, *H* = 10.486, *P* = 0.001). There was no significant difference between men and women in terms of how much they felt each of the remaining 13 words described themselves (see Table 2).

**Table 2.** Gendered personality traits.

	Gender	*N*	Mean rank	Kruskal-Wallis H	Asymptotic significance (two-tailed)^a^
Decisive (*N* = 171)	Woman	120	78.43	10.486	**0.001**
	Man	51	103.81		
Competitive (*N* = 171)	Woman	120	81.78	3.360	0.067
	Man	51	95.92		
Confident (*N* = 171)	Woman	120	82.56	2.281	0.131
	Man	51	94.09		
Leader (*N* = 171)	Woman	120	83.21	1.459	0.227
	Man	51	92.57		
Aggressive (*N* = 129)	Woman	89	62.96	1.075	0.300
	Man	40	69.55		
Tough (*N* = 170)	Woman	119	87.85	1.073	0.300
	Man	51	80.02		
Independent (*N* = 171)	Woman	120	88.32	1.045	0.307
	Man	51	80.54		
Determined (*N* = 170)	Woman	119	83.65	0.704	0.401
	Man	51	89.81		
Emotional (*N* = 171)	Woman	120	91.95	6.780	**0.009**
	Man	51	72.01		
Nurturing (*N* = 170)	Woman	119	90.89	5.432	**0.020**
	Man	51	72.91		
Giving (*N* = 171)	Woman	120	89.25	2.077	0.149
	Man	51	78.35		
Gentle (*N* = 171)	Woman	120	82.80	1.940	0.164
	Man	51	93.53		
Sensitive (*N* = 171)	Woman	120	88.83	1.479	0.224
	Man	51	79.35		
Accepting (*N* = 171)	Woman	120	87.12	0.262	0.609
	Man	51	83.37		
Patient (*N* = 171)	Woman	120	85.05	0.160	0.689
	Man	51	88.24		
Weak (*N* = 129)	Woman	89	64.94	0.001	0.978
	Man	40	65.13		

^a^Traits with significant differences (*P* < 0.05) are shown in bold.

### Gendered pain expectation

**Figure 1.**
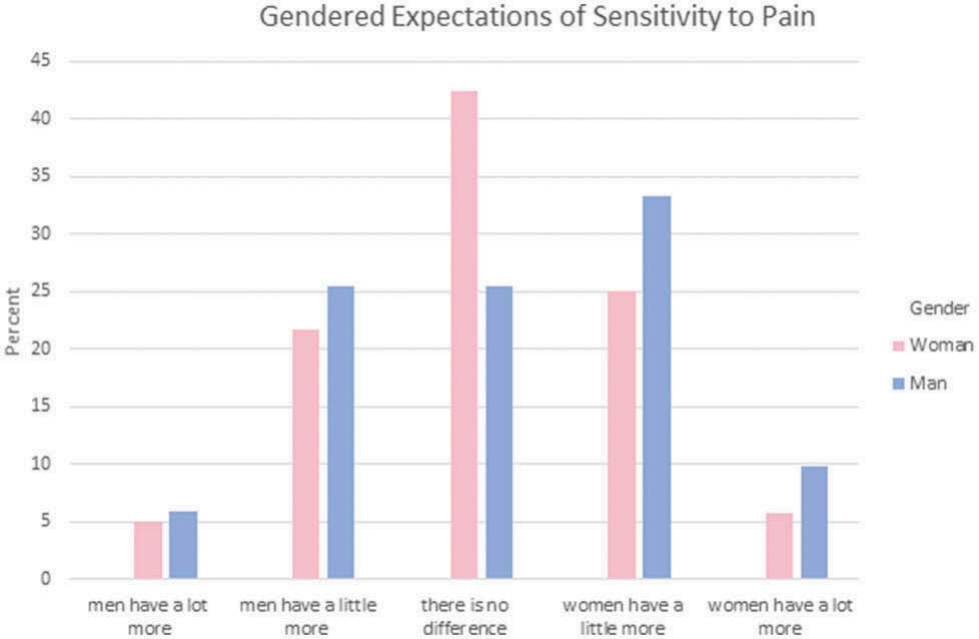
Gendered expectations of sensitivity to pain.

**Figure 2.**
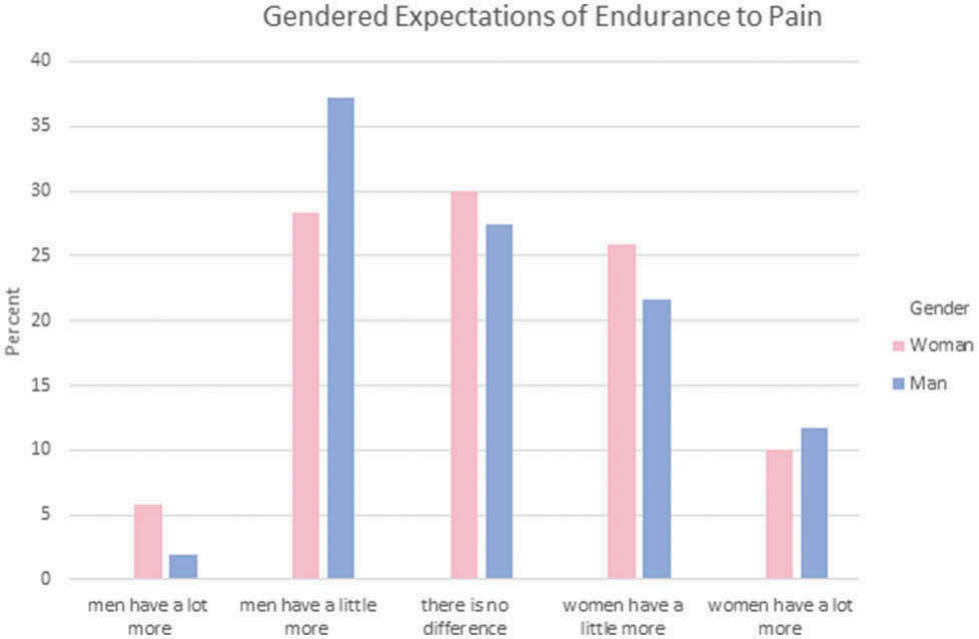
Gendered expectations of endurance to pain.

**Figure 3.**
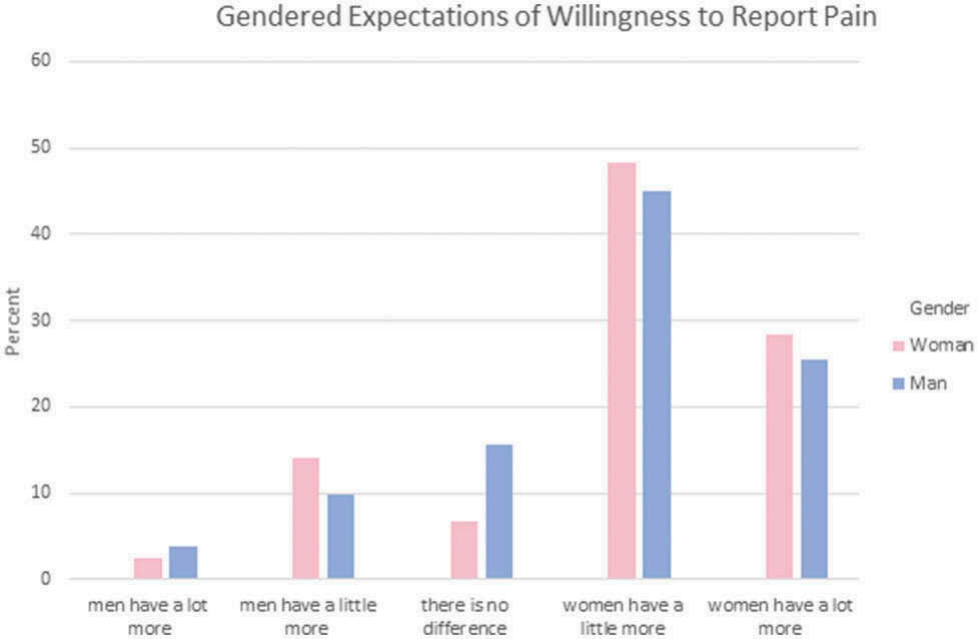
Gendered expectations of willingness to report pain.

Participants were asked to answer a set of three questions regarding how they feel each gender experiences different aspects of pain. These areas reflect areas where gendered expectations are known to occur. Specifically, they indicated which gender has more (1) sensitivity to pain; that is, the amount of injury or time required to cause pain (see Figure 1); (2) endurance to pain; that is, how much time passes before the person will seek relief from the pain (see Figure 2); and (3) willingness to report pain; that is, how willing men or women are to report their pain (see Figure 3). Responses were varied in terms of PT students’ perceptions of which gender has higher sensitivity and endurance to pain. However, the majority (75%) of men and women PT students felt that women were more willing to report pain compared to men.

Chi-square analysis was performed to explore whether gender differences exist in PT students’ expectations of pain sensitivity, pain endurance, or pain reporting among men and women. There were no statistically significant differences in terms of men and women PT students’ gender-related expectations of pain for a typical man and a typical woman in regard to pain sensitivity, χ^2^(4) = 4.757, *P* = 0.313, pain endurance, χ^2^(4) = 2.517, *P* = 0.642, or pain reporting, χ^2^(4) = 4.073, *P* = 0.396.

### Pain trait

**Figure 4.**
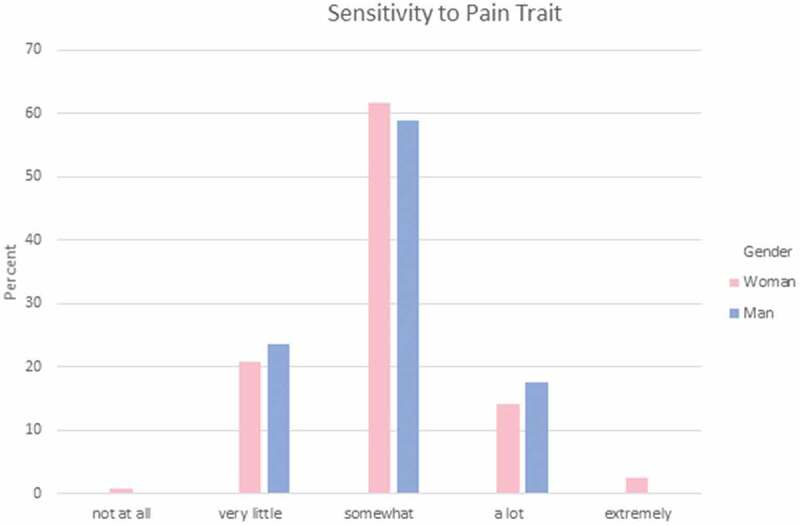
Sensitivity to pain trait.

**Figure 5.**
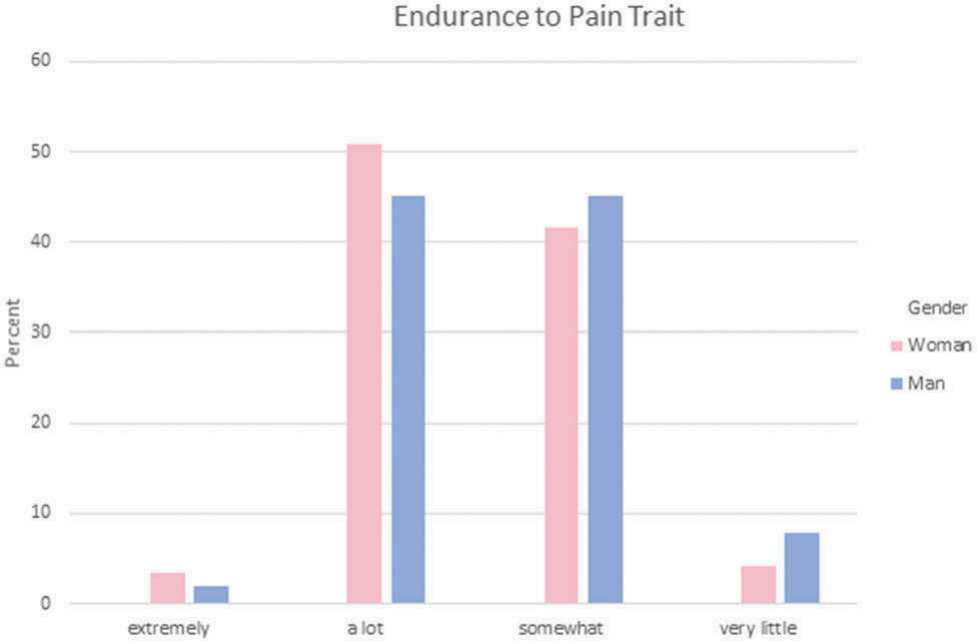
Endurance to pain trait.

**Figure 6.**
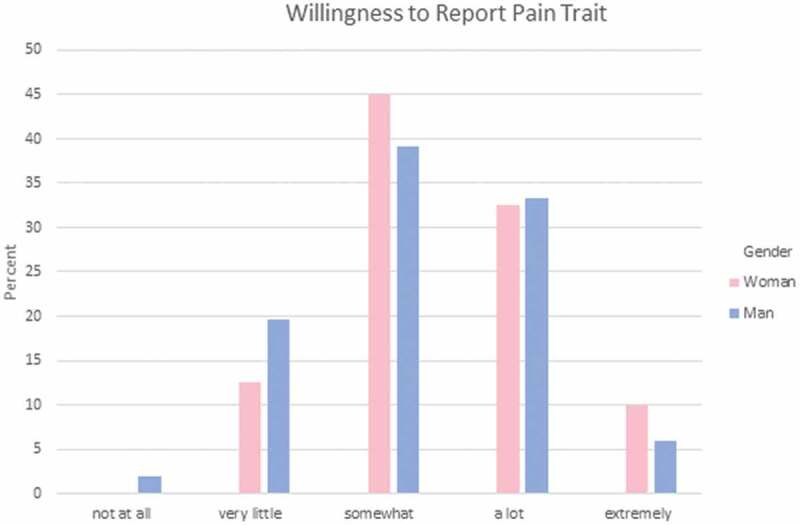
Willingness to report pain trait.

Each student was asked to rate their own sensitivity to pain, endurance for pain, and willingness to report pain with the following options: *not at all, very little, somewhat, a lot*, and *extremely*. The majority (61%) of PT students indicated that they were somewhat sensitive to pain (see Figure 4), 49% felt that they can endure pain a lot (see Figure 5), and 43% were somewhat willing to report pain (see Figure 6).

Kruskal-Wallis analysis was performed to examine whether the gender of PT students was significantly related to their self-reports of pain experience expectation. There were no statistically significant differences in self-reported sensitivity to pain, pain endurance, or willingness to report pain (see Table 3)

**Table 3.** Pain trait.

	Gender	*N*	Mean rank	Kruskal-Wallis H	Asymptotic significance (two-tailed)
Sensitivity to pain (*N* = 171)	Woman	120	86.26	0.015	0.903
	Man	51	85.38		
Endurance to pain (*N* = 171)	Woman	120	83.70	1.077	0.299
	Man	51	91.40		
Endurance to pain (*N* = 171)	Woman	120	88.49	1.154	0.283
	Man	51	80.15		

### Gender expectations alignment

Lastly, students were asked to compare how they handle pain relative to a typical man and a typical woman. Overall, the majority of PT students felt that they were not different in terms of handling pain compared to a typical man (*n* = 89, 64%) or a typical woman (*n* = 95, 59%). There was no statistical significance in terms of participants’ gender and their expectations of how they manage pain in comparison to a typical man, χ^2^(2, *N* = 140) = 5.878, *P* = 0.053. Similarly, no statistical significance was found in regard to PT students’ gender and their expectations of how they manage pain compared to a typical woman, χ^2^(2, *N* = 160) = 2.844, *P* = 0.241.

## Discussion

This study suggests that men and women PT students are similar in terms of a number of gendered traits that could be considered relevant to their roles as clinicians.

The few differences that did occur were consistent with gendered role expectations, in that women PT students rated themselves higher than men in terms of the traits emotional and nurturing. This is consistent with women having greater caregiving roles in society. If women are more nurturing, this could have positive effects on their clinical interactions.^20^ Though nurturing is difficult to measure, two aspects of nurturing are empathy and caring, which have been studied. Empathy is positively associated with patient satisfaction, treatment compliance, positive therapeutic relationships, and health-related quality of life outcomes.^21–23^ Similarly, caring has been shown to have an impact on patient satisfaction, speed of recovery, and discharge.^24^ However, a caution in this finding is that given that this a gender role expectation for women it can be difficult to differentiate whether women are actually more nurturing or are reporting to align with societal expectations.^25^ Further, we cannot assume that being nurturing is always the best approach in the every patient interaction. de Vugt et al.^25^ noted that a patient management style that is highly nurturing may result in a “parent–child approach” whereby the patient is “no longer regarded as an equal, p. 88.” We know that empowerment is important for adherence and self-management, especially in chronic conditions, and nurturing has to consider this balance.

In contrast, men rated themselves as more decisive than women, which is consistent with gendered expectations of men to be more focused on work, leadership, and goals. The fact that men rate themselves more highly on decisiveness might suggest that when it comes to making decisions about diagnosis or case management, men might feel more confident in these skills. Decisiveness has been shown to be a core competency for health care professionals.^26^ Though nurturing may be a positive trait in patient interaction, there is also some discussion in the literature about the positive aspects of a decisive approach.^26,27^ Studies have shown that patients appreciate decisive and “results-oriented” communication and that this instills confidence in patients.^26,27^ For patients who struggle to understand their illness or health interventions, a decisive approach can relieve anxiety. Further decisiveness may be protective for health care providers, because more decisive physicians experience less stress.^28^ Conversely, decisiveness may manifest as being paternalistic or nonlistening if the provider reaches a conclusion or recommendation more quickly than the patient is comfortable with or if the clinician interrupts the patient.

Decisiveness and nurturing each have potentially positive or negative effects, depending on how they are operationalized. Further, it is possible that each is operationalized or perceived differently on the basis of the gender of the clinician and patient. Understanding gendered traits and interactions and explicit discussion of these in training may be a mechanism for improved patient interactions.

Though previous studies have examined how gender and professions may influence pain-related expectations,^29^ no studies have examined the pain-related expectations of men and women PTs. In terms of gender-related expectations of pain in typical men and women, findings indicate that there are no gender differences in PT students’ expectations of pain sensitivity, endurance, and willingness to report in typical men and women. This shows that both men and women PT students share similar views regarding patients’ sensitivity, endurance, and willingness to report pain. However, results indicate that a majority of PT students believed that women are more willing to report pain.^8,9,29^ This is consistent with data on pain reporting; for example, a study by Wijnhoven et al.^30^ found that women reported more contact with health providers and more health care use than men. This has also been discussed as reluctance among men to seek care. Among health professionals, it may be that their expectations of pain reporting are based on the evidence or their clinical experiences, but both are influenced by societal norms. Gender norms and socialization tend to encourage men to be “tough” and stoic, which might partially explain underreporting of pain, and encouraging women to be sensitive and communicative may support greater pain reporting.^10^ A study by Levine and De Simone^31^ found that men reported significantly less pain in front of a female experimenter compared to a male experimenter, again indicating that gender traits and gender interactions may both affect clinical interactions. Gender differences are difficult to deconstruct from sex differences,^1^ because there are sex differences in pain sensitivity and endurance^7,32,33^ that may confound the relationships ascribed to gender. This creates a challenge for clinicians because understanding the influence of biology and social predictors of pain and response to interventions is important. Further, clinicians must take the spectrum of sex and gender into account, which means that individual differences may be larger than group differences, because generalizations can lead to biases. It is important to ensure that societal gender norms do not adversely impact how clinicians behave toward patients or their decisions regarding pain management.^29,34^ Clinicians should avoid reinforcing gender stereotypes, be self-reflective about the potential for gender bias in their own practice, and be aware of the potential for sex and/or gender to influence how patients present or respond to interventions.

We found no significant gender differences in sensitivity, endurance, and willingness to report pain amongst PT students. Pool et al.^35^ found evidence to support the idea that pain tolerance is dynamic and strongly correlated with a subject’s identification with gender norms, with men that identified strongly with typical male roles being more pain tolerant and men who did not identify with those beliefs having the same pain tolerance levels as women. Thus, our findings of similar pain expectations in PT students align with the similarity in their gendered traits. The lack of strong identification with stereotypical gender norms may be indicative of changing gender norms. For example, a 2011 report by the International Center for Research on Women found that the younger generation of men were more likely to carry out domestic duties, take paternity leave, and have more “gender-equitable” attitudes.^36^ Additionally, the similarity in gender traits in PT students may reflect their affinity to a caring profession or their training. This may also partially explain the similarity between men and women trainees with respect to gendered pain expectations.

In terms of gender expectations alignment, both men and women felt that they were no different in terms of how they handle pain compared to a typical man or a typical woman. Additionally, no significant gender differences were found. These findings suggest that men PT students do not see themselves as being more capable of handling pain compared to typical women. These findings differ from results of previous studies^8,11^ that found that men rated themselves to be less sensitive to pain^8,11^ and have higher endurance^11^ compared to typical women. One possible explanation is that our sample consisted of PT students who have had substantial clinical interactions with patients with a variety of painful conditions.

## Study limitations

Because our data are based on a self-reported survey, it is subject to social desirability bias. We tried to minimize this by making survey responses anonymous. We cannot evaluate the extent to which our sample represents the larger pool of PTs because we are uncertain how many PT students received the invitation to participate in the study. The sample included a disproportionate number of women PT students; however, the sample is representative because over 75% of all physiotherapists in Canada are female.^37^ The trainees were nearing completion of their clinical training program, and we did not assess how gender and pain perceptions varied over their training or based on their clinical experience. Most of the participants were from Western University, and this could limit generalizability. Finally, the survey was distributed in multiple ways (in class, through email and social media); however, we do not believe that the distribution method had an impact on participants’ responses.

## Future research

Although this study provides new information about PT students’ gendered pain beliefs, it is preliminary in nature due to inherent sampling and survey design limitations. Future studies and different research designs are needed. Qualitative studies that explore gender perceptions and experiences and models of how gender and related expectations of the clinician and the patient affects outcomes have the potential to improve patient experiences and outcomes. Future studies may want to investigate how gender and gender expectations influence pain sensitivity, endurance, and willingness to report pain in different pain populations, cultural groups, or age groups. Although gender bias did not seem to be a major issue, more in-depth exploration of gender issues and related pain expectations may help clinicians provide more patient-centered and unbiased pain rehabilitation.^33^ Additionally, studies on patient perceptions of PTs’ gender and pain experience/reporting are warranted. Given the physically intimate nature of PT and the emphasis on pain management, it is important that we examine pain reporting behavior among patients and how they differ based on PTs’ gender.

## Conclusion

Women nearing completion of their PT training self-identify as being more emotional and nurturing than men. In contrast, men in PT training identify with being more decisive., There were no significant difference on 13 other traits in the degree to which men and women PT students identify with gendered personality traits, indicating that men and women PT students demonstrate similar gender profiles. Men and women PT students have similar gender pain expectations, including that women are more likely to report pain. Men and women both had similar self-evaluations of how they handle and experience pain themselves.
